# Oxidative Dimerization of PHD2 is Responsible for its Inactivation and Contributes to Metabolic Reprogramming via HIF-1α Activation

**DOI:** 10.1038/srep18928

**Published:** 2016-01-07

**Authors:** Gibok Lee, Hyung-Sik Won, Yoon-Mi Lee, Jae-Wan Choi, Taek-In Oh, Jeong-Hwa Jang, Dong-Kug Choi, Beong-Ou Lim, Young Jun Kim, Jong-Wan Park, Pere Puigserver, Ji-Hong Lim

**Affiliations:** 1 Department of Biomedical Chemistry, College of Biomedical & Health Science, Konkuk University, Chungju 380-701, Chungbuk, Republic of Korea; 2 Department of Biotechnology, College of Biomedical & Health Science, Konkuk University, Chungju 380-701, Chungbuk, Republic of Korea; 3 Department of Food Bioscience, College of Biomedical & Health Science, Konkuk University, Chungju 380-701, Chungbuk, Republic of Korea; 4 Department of Pharmacology, Seoul National University, College of Medicine, Seoul 110-799, Republic of Korea; 5 Department of Cancer Biology, Dana-Farber Cancer Institute; Department of Cell Biology, Harvard Medical School, Boston, Massachusetts, USA

## Abstract

Prolyl hydroxylase domain protein 2 (PHD2) belongs to an evolutionarily conserved superfamily of 2-oxoglutarate and Fe(II)-dependent dioxygenases that mediates homeostatic responses to oxygen deprivation by mediating hypoxia-inducible factor-1α (HIF-1α) hydroxylation and degradation. Although oxidative stress contributes to the inactivation of PHD2, the precise molecular mechanism of PHD2 inactivation independent of the levels of co-factors is not understood. Here, we identified disulfide bond-mediated PHD2 homo-dimer formation in response to oxidative stress caused by oxidizing agents and oncogenic H-ras^V12^ signalling. Cysteine residues in the double-stranded β-helix fold that constitutes the catalytic site of PHD isoforms appeared responsible for the oxidative dimerization. Furthermore, we demonstrated that disulfide bond-mediated PHD2 dimerization is associated with the stabilization and activation of HIF-1α under oxidative stress. Oncogenic H-ras^V12^ signalling facilitates the accumulation of HIF-1α in the nucleus and promotes aerobic glycolysis and lactate production. Moreover, oncogenic H-ras^V12^ does not trigger aerobic glycolysis in antioxidant-treated or PHD2 knocked-down cells, suggesting the participation of the ROS-mediated PHD2 inactivation in the oncogenic H-ras^V12^-mediated metabolic reprogramming. We provide here a better understanding of the mechanism by which disulfide bond-mediated PHD2 dimerization and inactivation result in the activation of HIF-1α and aerobic glycolysis in response to oxidative stress.

Under aerobic conditions, prolyl hydroxylase domain proteins (PHDs) induce the hydroxylation of HIF-α on its oxygen-dependent degradation domain (ODDD), which results in its recognition by von Hippel-Lindau protein (pVHL) as an E3 ubiquitin ligase and subsequent degradation^1^,^2^,^3^. However, under anaerobic condition, HIF-1α accumulates and dimerizes with HIF-1β, and then translocates into the nucleus and transcriptionally activates target genes, including those involved in angiogenesis and glycolytic energy metabolism^4^,^5^. Additionally, HIF-1α can be stabilized and activated under aerobic conditions by oncogenic signalling pathways, including phosphatidylinositol-3 kinase (PI-3K)^6^,^7^, Ras^8^,^9^, and by mutations in tumour suppressors such as pVHL^10^ and succinate dehydrogenase (SDH)^11^, and by elevated intracellular reactive oxygen species (ROS) derived from multiple sources such as mitochondrial dysfunction^12^,^13^ and NADPH oxidase functioning^14^,^15^.

Several reports have suggested that elevated ROS levels lead to PHD2 inactivation and subsequently to HIF-α stabilization by regulating the levels of ascorbate, ferrous iron, or Krebs cycle intermediates^16^,^17^,^18^,^19^. Although, oxygen and co-factors limitation can regulate PHD2 enzymatic activity, it seems unlikely that this is only determinant of HIF-α stabilization and activation in the complex environment of the cell. According to a recent hypothesis, formation of inter or intramolecular disulfide bond in the cysteine residue of PHD2 may affect its enzymatic function^20^. However, whether PHD2 is modified through disulfide bond formation under oxidative stress is unknown.

Proliferating cancer cells compared to normal differentiated cells exhibit different metabolic pathways to support their biomass synthesis such as amino acids, lipids, and nucleic acids. They show increased glucose uptake and lactate production^21^, known as Warburg effect, to support their high rates of proliferation. Several oncogenic pathways such as HIF-1α, c-Myc, PI-3K, and Ras play important roles in enhancing aerobic glycolysis and suppressing oxidative phosphorylation, thereby suggesting their involvement in the metabolic reprogramming of cancer cells^22^. Recent studies suggested that oncogenic transformation by H-ras^V12^ may alter cellular metabolism, including promotion of glucose flux and lactate production and increased generation of ROS^23^,^24^,^25^. Although oncogenic H-ras^V12^ signalling leads to metabolic reprogramming from oxidative phosphorylation to glycolysis in immortalized fibroblast by stabilizing and activating HIF-1α^8^,^9^, the underlying mechanism including the effects of redox status during the metabolic reprogramming remains poorly understood.

Here, we show that PHD2 dimerization and its inactivation caused by oncogenic H-ras^V12^-associated oxidative stress result in HIF-1α activation and consequent increased glucose flux and lactate production.

## Results

### Oxidative stress induces PHD2 dimerization through disulfide bond formation

**Figure 1:**
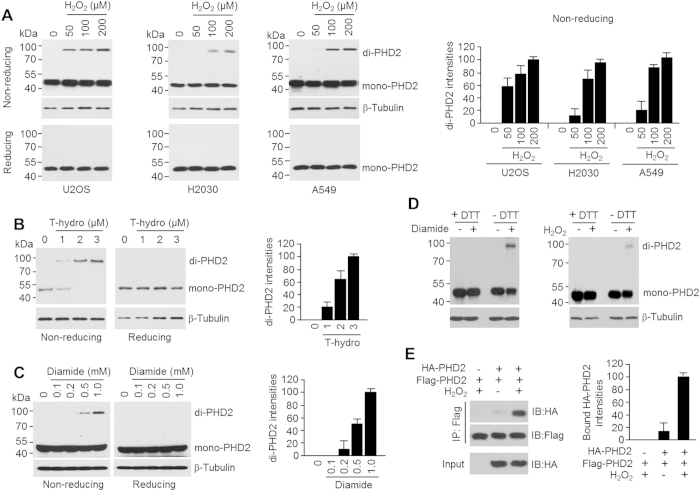
Oxidative stress leads to PHD2 homo-dimerization via disulfide bond formation. (**A**) Indicated concentration (50, 100, 200 μM) of H_2_O_2_ were treated in U2OS (osteosarcoma), H2030 (lung adenocarcinoma), and A549 (lung carcinoma) cancer cells for 1 h. Total protein was isolated using lysis buffer without reducing agents, and then lysates were subjected into SDS-PAGE with reducing or non-reducing sample buffer (*n* = 3). Detail procedure is explained in the method and materials section. (**B**) U2OS cells were incubated with different concentrations (1, 2, 3 μM) of T-hydro for 30 min, and then total protein was subjected into SDS-PAGE with reducing or non-reducing sample buffer (*n* = 3). (**C**) U2OS cells were incubated with different concentrations (0.1, 0.2, 0.5, 1 mM) of diamide for 30 min, and then total protein was subjected into SDS-PAGE with reducing or non-reducing sample buffer (*n* = 3). (**D**) Diamide (1 mM) or H_2_O_2_ (0.2 mM) was introduced into U2OS cells for 30`min, and then total cell lysates were further incubated with or without 100 mM of dithiothreitol (DTT). (**E**) HA-tagged PHD2 (0.5 μg/ml) and/or Flag-tagged PHD2 (0.5 μg/ml) was transiently transfected into U2OS cells. 24 h post-transfection, cells were incubated with 0.2 mM of H_2_O_2_ for 2 h, and then PHD2 homo-dimerization in native condition was detected by co-immunoprecipitation assay.

Because a specific disulfide bond-mediated dimerization under oxidative stress was observed for many proteins^26^, it was highly intriguing whether PHD2 dimerization can be also an oxidant treatment. Using non-reducing gel electrophoresis, we successfully detected the PHD2 dimerization in various cancer cells including U2OS, H2030, and A549 upon the 50~200 μM of hydrogen peroxide (Fig. 1A). In addition, we also found that 50 μM of hydrogen peroxide enough to increase PHD2 dimerization especially in U2OS cells. Furthermore, use of reducing agent, β-mercaptoethanol (β-ME), revoked hydrogen peroxide-induced PHD2 dimerization. Additionally, we tested whether distinct types of oxidants also induce PHD2 dimerization in cultured cells. Figure 1B shows that *tert*-butyl hydroperoxide (T-hydro), an organic peroxide, dramatically induced PHD2 dimerization in non-reducing gel electrophoresis. We further examined PHD2 dimerization by SDS-PAGE in the absence or presence of diamide, a thiol-oxidizing compound, under non-reducing or reducing conditions. PHD2 dimer was detected after diamide pretreatment in non-reducing gel electrophoresis compared to the β-ME containing gel electrophoresis (Fig. 1C). When the lysates of the oxidant (hydrogen peroxide or diamide)-treated cells were incubated with dithiothreitol (DTT), a strong reductant that causes reduction of a typical disulfide bond, PHD2 dimers were not detected even in the nonreducing SDS-PAGE (Fig. 1D), suggesting the dissociation of the oxidant-induced PHD2 dimers into monomers by removing the intermolecular disulfide bonds. We further examined whether PHD2 formed a homo-dimer under oxidative stress. Oxidation-induced molecular association of Flag-tagged PHD2 and HA-tagged PHD2 was observed in the co-immunoprecipitation assay, confirming homo-dimeric organization of the oxidized PHD2, as shown Fig. 1E. Together, these results suggest that oxidative stress leads to disulfide bond-mediated PHD2 homo-dimerization.

### Oncogenic H-ras^V12^ signalling leads to PHD2 homo-dimerization

**Figure 2:**
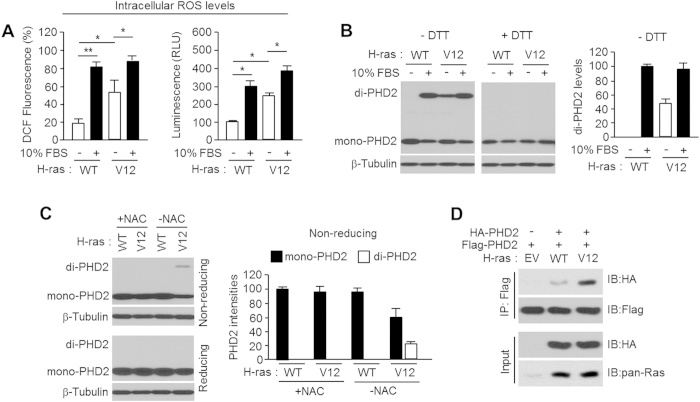
Oncogenic H-ras^V12^ leads to PHD2 homo-dimerization through oxidative stress. (**A**) Intracellular reactive oxygen species (ROS) level was measured using an oxidation-sensitive probe DCF-DA or luciferase-based ROS-Glo^TM^ H_2_O_2_ assay kit. Values represent mean ± SD of independent experiments performed three times; *p < 0.05 and **p < 0.01. (**B**) Wild type or G12V mutant of H-ras stably expressing U2OS cells were incubated without foetal bovine serum (FBS) for 6 h. After serum starvation, cells were incubated with or without 10% of FBS for 3 h. Total cell lysates were further incubated with or without 100 mM of dithiothreitol (DTT) *in vitro*, and then DTT protein samples were subjected into SDS-PAGE. Dimer form of PHD2 intensity is represented as a bar graph using mean ± SD of three independent experiments. (**C**) H-ras^WT^ or H-ras^V12^ overexpressing U2OS cells were incubated for 16 h with or without 3 mM of NAC. PHD2 dimerization was detected by immunoblotting as indicated. (**D**) HA-tagged PHD2 (0.5 μg/ml) and/or Flag-tagged PHD2 (0.5 μg/ml) was transiently transfected into U2OS cells as indicated, and then PHD2 homo-dimerization in native condition was detected by co-immunoprecipitation assay.

Oncogenic growth signalling pathways such as H-ras^V12^ and Bcr-Abl tyrosine kinase increases intracellular ROS levels. Several cancer cells with mutations in their growth signalling pathways exhibit high levels of intracellular ROS^27^,^28^,^29^. We generated hyperactive form of H-ras^V12^-overexpressing U2OS cells to determine whether hyper-activation of the oncogenic growth signalling-mediated ROS induces PHD2 dimerization. Consistent with previous reports^29^, intracellular ROS levels were significantly increased by the hyperactive H-ras^V12^ overexpression in U2OS cells (Fig. 2A). Figure 2B shows that 10% of foetal bovine serum (FBS) induced PHD2 dimerization and reduced monomer formation of PHD2 in U2OS cells. Interestingly, PHD2 dimerization increased in the hyperactive H-ras^V12^-overexpressing U2OS cells even in the absence of FBS. Oncogenic growth signalling-induced PHD2 dimerization ceased upon treatment with DTT. To investigate whether the oncogenic growth signaling-induced intracellular ROS is required for PHD2 dimerization, we examined PHD2 dimerization in the absence or presence of an antioxidant, *N*-acetyl-l-cysteine (NAC), in the H-ras^V12^-overexpressing U2OS cells. Figure 2C shows that PHD2 dimerization induced by H-ras^V12^ overexpression ceased upon treatment with NAC. Increasing homo-dimerization of PHD2 by H-ras^V12^ overexpression was confirmed by co-immunoprecipitation assay (Fig. 2D). These results revealed that oncogenic H-ras^V12^ signalling promotes disulfide bond-mediated PHD2 homo-dimerization.

### ROS inhibits PHD2 enzymatic activity and oncogenic H-ras^V12^ signalling stabilizes HIF-1α

**Figure 3:**
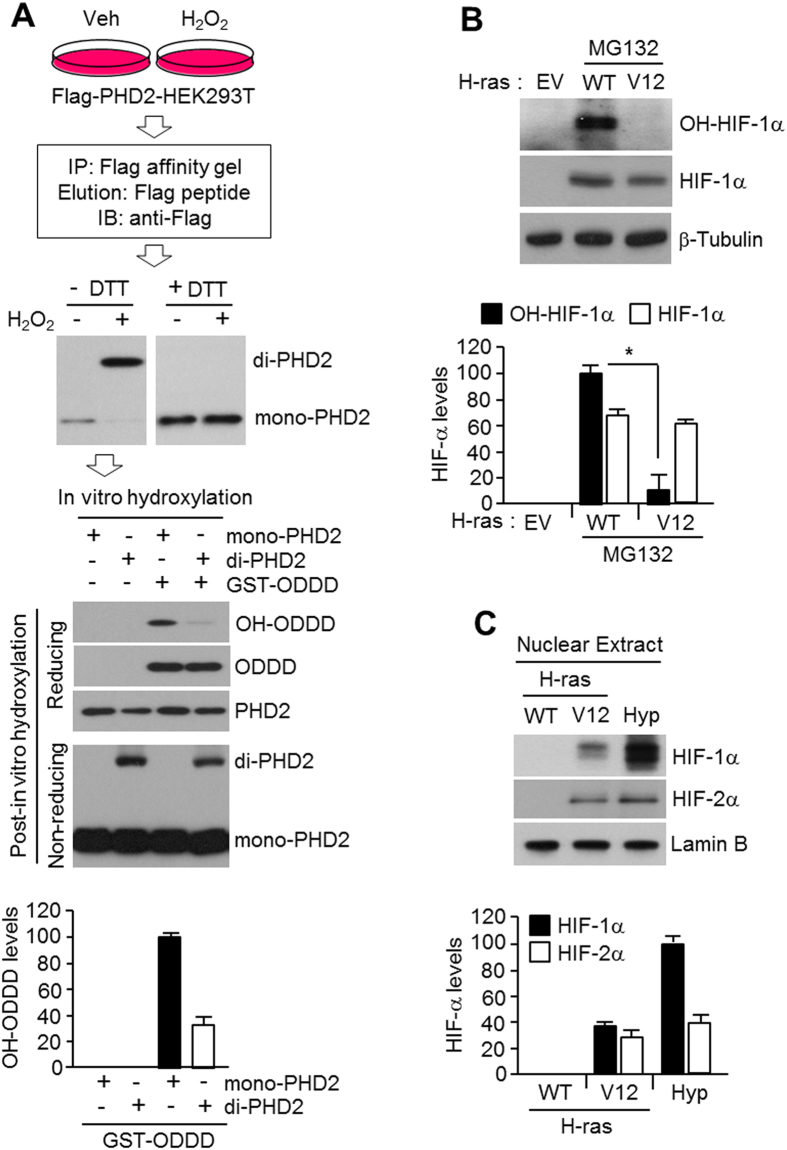
PHD2 inactivation and HIF-1α accumulation by H-ras^V12^. (**A**) To obtain monomer or dimer form of PHD2 from HEK293T cells, Flag-tagged PHD2 stably expressing HEK293T cells were incubated for 1 h in the absence or presence of 200 μM of H_2_O_2_. After pre-incubation, total lysates were incubated with Flag-affinity gel for 1 h at 4 °C, and then Flag-tagged PHD2 proteins were eluted using Flag-peptide. The eluted Flag-PHD2 was subjected into SDS-PAGE with reducing or non-reducing gel electrophoresis. For *in vitro* hydroxylation assay, bacterial expressed and purified ODDD was incubated with monomer or dimer form of Flag-PHD2 produced in HEK293T cells in the absence or presence of H_2_O_2_. After reaction, protein samples were subjected into SDS-PAGE with reducing or non-reducing gel electrophoresis (*n* = 3). (**B**) Endogenous hydroxylated HIF-1α levels in H-ras^WT^ or H-ras^V12^ overexpressing U2OS cells were detected by immunoblotting using Pro564(OH)-HIF-1α antibody (*n* = 3). (**C**) Accumulated nuclear HIF-1α or HIF-2α in H-ras^WT^ or H-ras^V12^ overexpressing U2OS cells were detected by immunoblotting using HIF-1α or HIF-2α antibody (*n* = 3).

What is a biological function of ROS-mediated PHD2 dimerization ? To answer this question, we determined PHD2 enzymatic activity using monomer or dimer form of PHD2 purified from HEK293T cells cultured in the absence or presence of hydrogen peroxide. Purified PHD2 from hydrogen peroxide-treated HEK293T cells exhibited homo-dimer form, and lacked monomer form in the absence of DTT. Moreover, oxidation-induced PHD2 dimerization ceased in the presence of DTT (Fig. 3A). Because the oxygen-dependent degradation domain (ODDD) of HIF-1α is a PHD2 target substrate^30^, we next investigated whether the disulfide bond-mediated PHD2 dimerization affects prolyl hydroxylation on ODDD using HEK293T cells derived monomer or dimer form of PHD2 proteins. Figure 3A shows that monomer form of PHD2 strongly induces HIF-1α ODDD hydroxylation. On the other hand, hydroxylation of ODDD was dramatically decreased by approximately 60% by dimer form of PHD2 derived from hydrogen peroxide-treated HEK293T cells, indicating that hydrogen peroxide-induced dimer form of PHD2 is less active than monomer form of PHD2 on ODDD hydroxylation. We evaluated hydroxylated HIF-1α levels in H-ras^V12^-overexpressing U2OS cells to determine whether oncogenic H-ras^V12^ inhibits HIF-1α hydroxylation. Figure 3B shows that oncogenic H-ras^V12^ expression resulted in reduced levels of hydroxylated HIF-1α in the presence of proteasome inhibitor, MG132, under normoxic condition. Additionally, nuclear accumulated HIF-1α and HIF-2α levels were increased in H-ras^V12^-overexpressing U2OS cells (Fig. 3C). These results indicate that oxidative stress results in elevated HIF-1α and HIF-2α levels due to PHD2 inactivation by disulfide bond-mediated homo-dimerization.

### ROS induced by oncogenic H-ras^V12^ activates HIF-1α dependently of PHD2

**Figure 4:**
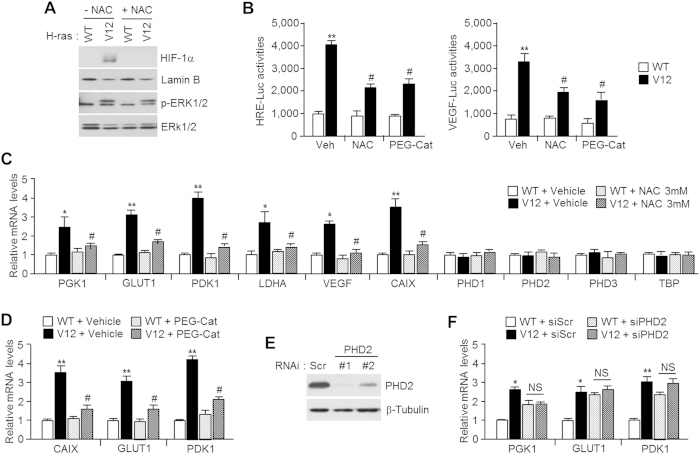
Inactive PHD2 caused by reactive oxygen species (ROS) participate in HIF-1α target glycolytic gene expression. (**A**) H-ras^WT^ or H-ras^V12^ expressing U2OS cells were incubated in the absence or presence of 3 mM of NAC for 16 h, and then HIF-1α levels were detected by immunoblotting. (**B**) Cells were transiently transfected with indicated reporter plasmids in H-ras^WT^ or H-ras^V12^ stably expressing U2OS cells. Transfected cells were further incubated with 3 mM of NAC or 50 units/ml of PEG-conjugated catalase for 24 h. Luciferase activity was measured using a luminometer, and β-gal assay was performed to normalize transfection efficiencies. Values represent mean ± SD of independent experiments performed three times; **p < 0.01 versus H-ras^WT^ vehicle and ^#^*p* < 0.05 versus H-ras^V12^ vehicle. (**C**) H-ras^WT^ or H-ras^V12^ stably expressing U2OS cells were cultured in the absence or presence of 3 mM of NAC for 48 h, and then HIF-1α target genes or PHD isoforms were assessed by quantitative PCR. Values represent mean ± SD of three independent experiments performed in triplicate; *p < 0.05, **p < 0.01 versus H-ras^WT^ vehicle, and ^#^*p* < 0.05 versus H-ras^V12^ vehicle. (**D**) H-ras^WT^ or H-ras^V12^ stably expressing U2OS cells were cultured in the absence or presence of 50 units/ml of PEG-conjugated catalase for 48 h, and then HIF-1α target genes were assessed by quantitative PCR. Values represent mean ± SD of three independent experiments performed in triplicate; **p < 0.01 versus H-ras^WT^ vehicle, and ^#^*p* < 0.05 versus H-ras^V12^ vehicle. (**E**) U2OS cells were transfected with 40 nmol/L small interfering RNA (siRNA) targeting PHD2, and then endogenous PHD2 levels were detected by immunoblotting. (**F**) H-ras^WT^ or H-ras^V12^ stably expressing U2OS cells were transiently transfected with 40 nmol/L of siRNA against PHD2, and then cells were further incubated for 48 h. After transfection and incubation, HIF-1α target genes were assessed by quantitative PCR. Values represent mean ± SD of three independent experiments performed in triplicate; *p < 0.05 and **p < 0.01.

NAC treatment impaired nuclear accumulated HIF-1α in H-ras^V12^-overexpressing U2OS cells (Fig. 4A), suggesting that increased intracellular ROS level is necessary for H-ras^V12^-mediated HIF-1α nuclear accumulation. To determine the physiological relevance of PHD2 dimerization caused by oxidative stress, we assessed HIF-1α activity in H-ras^V12^-overexpressing U2OS cells. Figure 4B shows that HIF-1α activity was strongly enhanced by H-ras^V12^ overexpression, but this attenuated by addition of NAC or polyethylene glycol (PEG)-conjugated catalase. Likewise, HIF-1α target genes related to glycolysis, including phosphoglycerate kinase 1 (PGK1), glucose transporter 1 (GLUT1), pyruvate dehydrogenase kinase 1 (PDK1), and lactate dehydrogenase A (LDHA), were also up-regulated, but not PHD isoforms and TATA box binding protein (TBP) in H-ras^V12^-overexpressing U2OS cells and suppressed by NAC treatment (Fig. 4C). Although previous report shown that PHD isoforms are increased under hypoxia^31^, we cannot found the up-regulation of PHD isoforms in H-ras^V12^ expressing cells. This result suggests that H-ras^V12^-induced HIFα is not sufficient to increase PHD isoforms due to the distinction transcriptional mechanism regarding determination of target gene set between hypoxia and oncogenic signaling-induced HIFα. In addition, PEG-conjugated catalase also significantly blocked H-ras^V12^-induced HIF-1α target gene expression (Fig. 4D). To determine whether PHD2 is required for HIF-1α activation in H-ras^V12^-expressing cells, we genetically inhibited PHD2 expression using small interfering RNA (siRNA) (Fig. 4E) and compared the HIF-1α target gene expressions with wild-type and H-ras^V12^-expressing U2OS cells. Figure 4F shows that H-ras^V12^ does not promote HIF-1α target gene expression in PHD2 knocked-down cells, although the genetic suppression of PHD2 in the wild-type cells up-regulate the HIF-1α target gene expression. These results indicate that PHD2 is necessary for HIF-1α activation under oxidative stress caused by oncogenic H-ras^V12^ signalling.

### Oxidative stress caused by oncogenic H-ras^V12^ signalling leads to metabolic reprogramming by inhibiting PHD2 and stabilizing HIF-1α

**Figure 5:**
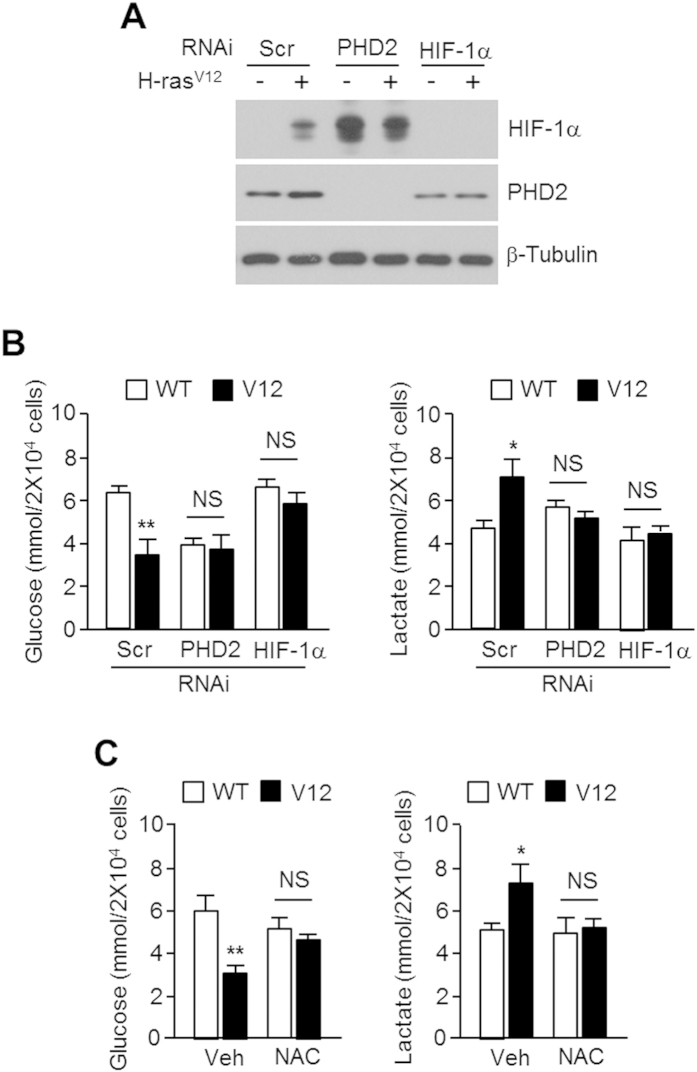
Oncogenic H-ras^V12^ enhances aerobic glycolysis by inhibiting PHD2 and activating HIF-1α. (**A**) H-ras^WT^ or H-ras^V12^ expressing U2OS cells were transiently transfected with indicated siRNA (40 nmol/L), and further incubated for 48 h. Protein levels were measured by immunoblotting. (**B,C**) Cultured medium was used to measure extracellular glucose and lactate levels as described in Methods section. Values of three independent experiments performed in duplicate; *p < 0.05 and **p < 0.01.

We determined whether disulfide bond-mediated PHD2 dimerization is crucial for HIF-1α activation and metabolic reprogramming induced by H-ras^V12^, as oncogenic H-ras^V12^ activates glycolysis via HIF-1α-mediated metabolic reprogramming in immortalized fibroblast cells^8^,^9^. Figure 5A shows that H-ras^V12^ expression induces HIF-1α, but is absent in PHD2 knocked-down cells, suggesting that PHD2 is required for HIF-1α activation by oncogenic H-ras^V12^ expression. To assess the role of ROS-mediated PHD2 dimerization during metabolic reprogramming through H-ras^V12^-mediated HIF-1α activation, we measured glucose utilization and lactate production in cultured medium. Glucose utilization and lactate production dramatically increased in oncogenic H-ras^V12^-expressing U2OS cells, but H-ras^V12^ did not affect metabolic reprogramming in PHD2 or HIF-1α knocked-down cells (Fig. 5B). Figure 5C reveals that NAC treatment completely blocked metabolic reprogramming (increased glucose utilization and lactate production) caused by oncogenic H-ras^V12^ expression. These results indicate that ROS-mediated PHD2 inactivation is a key step for H-ras^V12^-mediated HIF-1α activation and metabolic reprogramming in cancer cells.

### Cysteines in the DSBH region is required for the oxidative dimerization

**Figure 6:**
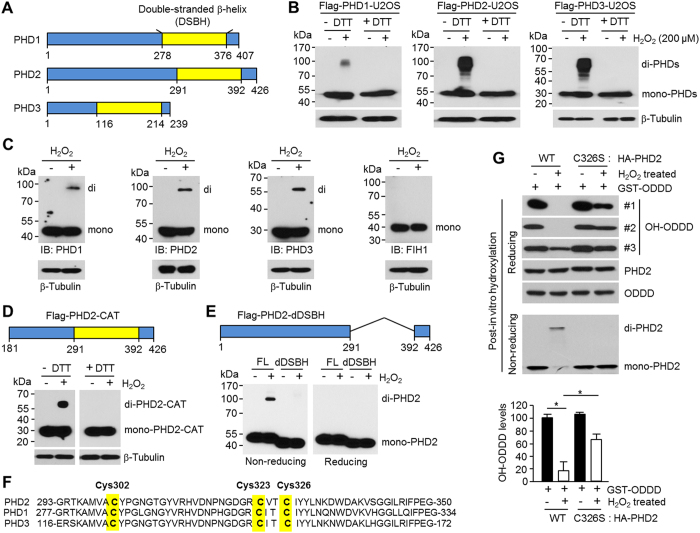
Cys326 in the DSBH region of PHD2 is involved to the PHD2 homo-dimerization upon hydrogen peroxide treatment. (**A**) Schematic diagram of protein domain structures of PHD isoforms. (**B**) Flag-tagged PHD1, 2, or 3 stably expressing U2OS cells were incubated with 200 μM of H_2_O_2_ for 1 h, and then total cell lysates were further incubated with or without 100 mM of DTT. Total protein was subjected into SDS-PAGE, and then indicated protein levels were measured by immunoblotting (*n* = 3). (**C**) H_2_O_2_ (200 μM) was introduced into U2OS cells for 1 h, and then total cell lysates were subjected into non-reducing SDS-PAGE. Indicated protein levels were measured by immunoblotting (*n* = 4). (**D**) Flag-tagged catalytic domain of PHD2 (Flag-PHD2-CAT) was transiently transfected into U2OS cells. 24 h post-transfection, cells were incubated with 200 μM of H_2_O_2_ for 1 h, and then total cell lysates were further incubated with or without 100 mM of DTT. Total protein was subjected into SDS-PAGE, and then indicated protein levels were measured by immunoblotting using anti-HA (*n* = 3). (**E**) DSBH region deleted Flag-PHD2 (Flag-PHD2-dDSBH) was transiently transfected into U2OS cells. 24 h post-transfection, cells were incubated with 200 μM of H_2_O_2_ for 1 h, and then total cell lysates were subjected into non-reducing or reducing SDS-PAGE. Indicated protein levels were measured by immunoblotting using anti-Flag (*n* = 3). (**F**) Sequence alignment of human PHD1, 2, and 3 proteins. The yellow bar denotes a conserved cysteine residue. (**G**) C326S or wild-type HA-PHD2 were transiently transfected into HEK293T cells, and then cells were incubated with 100 μM of H_2_O_2_ for 1 h. Total cell lysates were incubated with HA-affinity gel for 1 h, and then HA-bound PHD proteins were eluted using HA-peptide. For *in vitro* hydroxylation assay, bacterial expressed and purified ODDD was incubated with monomer or dimer form of HA-PHD2 produced in HEK293T cells in the absence or presence of H_2_O_2_. After reaction, protein samples were subjected into SDS-PAGE with reducing or non-reducing gel electrophoresis (*n* = 4).

To identify the critical region of PHD2 protein that is responsible for oxidative stress-induced dimerization, we first examined whether PHD1 and PHD3 (Fig. 6A in comparison), as well as PHD2, could also dimerize upon the hydrogen peroxide treatment. Figure 6B reveals that, like Flag-PHD2, the overexpressed Flag-PHD1 and PHD3 were also dimerized by 200 μM of hydrogen peroxide in the absence of DTT. Figure 6C confirms again that endogenous dimerization of PHD1, PHD2, and PHD3, but not the factor inhibiting HIF-1α (FIH1), was increased upon the hydrogen peroxide treatment. Moreover, this result indicates that PHD3 is more highly sensitive to the oxidative dimerization (Fig. 6B, C). As PHD3 is the shortest one of the three PHD isoforms, its mainly conserved double-stranded β-helix (DSBH) fold region, which comprises the 2-oxoglutarate (2-OG) and Fe^2+^ interacting, catalytic active site, occupies relatively higher portion of the whole protein, than in other isoforms^32^,^33^. Thus, we investigated whether the DSBH region is commonly required for homo-dimerization of PHD proteins. Figure 6D shows that the C-terminal catalytic domain of PHD2 (Flag-PHD2-CAT) that contained the DSBH dramatically dimerized upon hydrogen peroxide treatment in the absence of DTT. In sharp contrast, the DSBH-truncated PHD2 (Flag-PHD2-dDSBH) was not capable of oxidative dimerization (Fig. 6E), indicating that the homo-dimer formation of PHD isoforms could be critically mediated by the DSBH region. The DSBH region in PHD2 possesses three strictly conserved cysteines (Cys302, Cys323, and Cys326; Fig. 6F), which might be able to serve as potential requirements for the oxidative dimerization, through disulfide bond formation and/or by inducing a certain conformational change, upon oxidation. In addition, recent investigation by Chowdhury *et al.* has indicated that at least two of the three cysteines in the DSBH region of PHD2 could be modified by nitric oxide (NO) to form the S-nitrosylated cysteine, which also suggests plausible potentials of their sulfhydryl groups to readily react with oxidants, as well as NO^34^. In this context, we further investigated to identify the critical cysteine residue for homo-dimer formation of PHD isoforms. As results (Supplementary Fig. 2), significant attenuations of the oxidative dimerization were observed in the C302S and more profoundly in the C326S mutation, while the mutation at Cys328, which is located out of the DSBH, was still highly sensitive to the oxidative dimerization. Furthermore, we investigated whether oxidative stress could directly affect PHD2 enzymatic activity through oxidative homo-dimerization independently of iron oxidation. Figure 6G shows that C326S-PHD2 that purified from H_2_O_2_-treated HEK293T cells was not dimerized under non-reducing gel electrophoresis. Consistently, purified C326S-PHD2 unlikely dimer form enriched wild type-PHD2 in H_2_O_2_-treated HEK293T cells significantly increased ODDD hydroxylation *in vitro*. These results suggest that the cysteines in the DSBH region, particularly including Cys326, would be critically required for the disulfide bond-mediated PHDs homo-dimerization and its enzymatic activity regulation under oxidative stress.

## Discussion

Oxidizing conditions can induce disulfide bond formation in many cytosolic proteins, which can affect their biological function such as signal transduction and regulation of antioxidant system^26^. Several reports have shown that cytosolic proteins, including pyruvate kinase M2 (PKM2), phosphatase and tensin homolog (PTEN), NEMO, and protein tyrosine phosphatases (PTPs), are directly regulated by intracellular ROS through inter or intramolecular disulfide bond formation^36^,^37^,^38^,^39^. Although, several reports have shown that oxidizing ferrous iron inactivates PHD2 under oxidative stress^16^,^17^, it seems unlikely that this is the sole mechanism of PHD2 regulation. Interestingly, recent report has shown that T-hydro, organic peroxide, strongly inhibits FIH1-mediated HIF-1α N803 hydroxylation. Moreover, 1.5 μM of T-hydro significantly decreased HIF-1α P402 and P564 hydroxylation by approximately 50% in RCC4 cells, suggesting that oxidative stress could stabilize and activates HIF-1α by modulating its hydroxylation status^35^. Nevertheless, the precise mechanism underlying how oxidative stress controls HIF hydroxylase not understood. A possible hypothesis suggested that oxidative stress could change the disulfide bond structure of PHD2 to inhibit its enzymatic activity^20^,^40^. Here, we successfully determined disulfide bond-mediated homo-dimer formation of PHD isoforms, but not FIH1, in the presence of H_2_O_2_, T-hydro, diamide, or H-ras^V12^ expression, as a physiological oxidative stress condition *in vivo* and *in vitro*, which is similar to other redox sensitive cytosolic proteins such as NEMO^37^ and receptor protein-tyrosine phosphatase α (RPTPα)^39^. Consistent with previous observation for proteins, disulfide bond-mediated PHD2 dimerization ceased with reducing agent, β-ME or DTT. Additionally, HIF-1α hydroxylation assay revealed that the PHD2 dimer formed by H_2_O_2_ and H-ras^V12^ expression would be less active than its monomer form *in vitro* and *in vivo*. Therefore, these findings suggest that structural repositioning of PHD2 through disulfide bond-mediated dimerization is an important mechanism for regulating its enzymatic activity.

Cysteine residues have diverse roles in the structure, function, and regulation of proteins. They combine with the nearby cysteines to form inter- or intramolecular disulfide bonds, making this the primary route for oxidant-mediated disulfide bond formation^26^. Reversibly, oxidant-mediated inter- or intramolecular disulfide bond can be dissociated by reducing agents, including β-ME and DTT^37^,^39^. For instance, exposure to oxidizing conditions can induce NEMO homo-dimer formation through disulfide bond formation between Cys54-Cys54 and Cys347-Cys347^37^. In the present study, cysteine residues in the catalytic DSBH region also appeared to be responsible for the oxidative dimerization of PHD2. Particularly, the mutations at Cys326 led to a near complete loss of the observed oxidative dimerization. Unfortunately, however, the substrate- and/or inhibitor-free structure of PHD2 is not available yet. Thus, an underlying molecular model addressing how the cysteine oxidation can occur and induces the dimerization-mediated inactivation of PHD2 remains to be further investigated in line with a detailed structural study of the ligand-free PHD2 at both the reduced and oxidized states.

**Figure 7:**
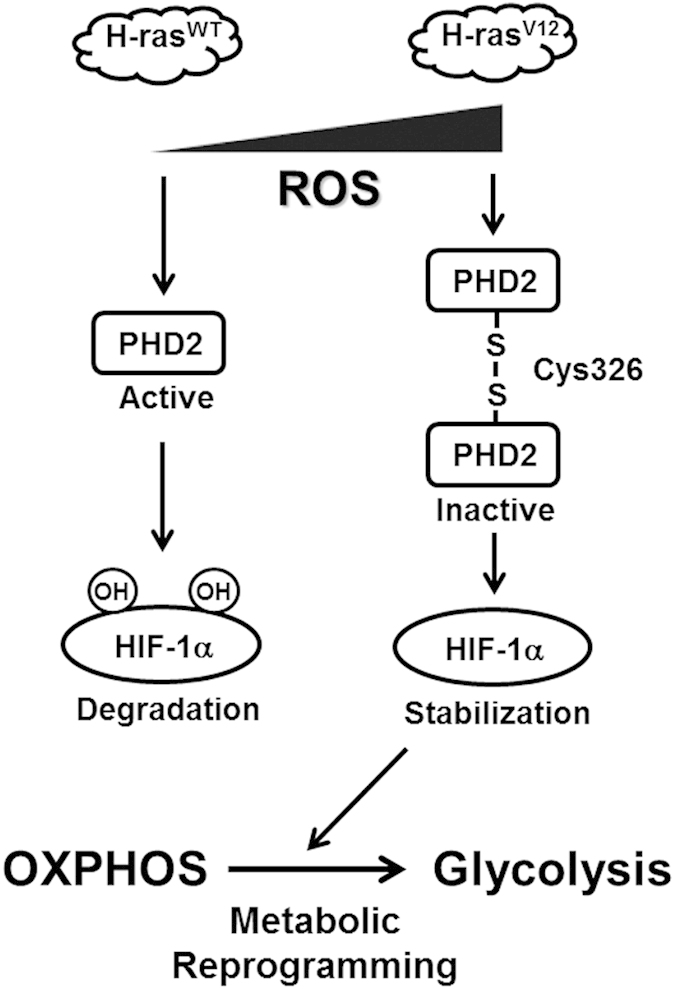
Proposed mechanism of PHD2 dimerization and inactivation leading to HIF-1α-mediated metabolic reprogramming under oncogenic H-ras^V12^ signalling-induced reactive oxygen species (ROS). Intracellular ROS levels are increased by oncogenic H-ras^V12^ transformation. Accumulated ROS level may leads to disulfide bond between Cys326-mediated PHD2 dimerization and inactivation results in increased aerobic glycolysis via HIF-1α stabilization.

Oncogenic growth signalling pathways such as PI-3K, Ras, and Wnt are critical for metabolic reprogramming from oxidative phosphorylation to glycolysis in cancer^22^,^41^. For example, increased glucose utility and lactate production have been observed in H-ras^V12^ overexpressing immortalized fibroblast, suggesting that oncogenic Ras signalling causes a global shift in the energy metabolism^8^,^9^. Here, we show that H-ras^V12^ elevates glycolytic flux and lactate production, also called Warburg effect, by inhibiting PHD2 activity and activating HIF-1α under aerobic condition. In addition, increased oxidative stress is commonly observed in proliferative cancer cells in abnormal microenvironment such as hypoxia and genetic alterations such as p53, PI-3K, Myc and Ras^22^. However, it is not clear whether oxidative stress could fine-tune metabolic flexibility in proliferative cancer cells. Our results showed that antioxidant, NAC, significantly inhibits enhanced glycolysis in oncogenic H-ras^V12^ expressing cells. These observations indicate that accumulation of ROS is required for metabolic reprogramming from oxidative phosphorylation to glycolysis caused by oncogenic H-ras^V12^ via PHD2 inactivation mediated by intermolecular disulfide bond between the Cys326 and HIF-1α activation (Fig. 7).

Together, these observations provide a novel mechanistic link between disulfide bond-mediated PHD2 dimerization and consequently HIF-1α stabilization upon oxidative stress in several cancer cells. The major findings include: (1) Oncogenic growth signalling-mediated ROS accumulation leads to PHD2 dimerization through disulfide bond formation; (2) PHD2 dimer induced by oxidative stress is functionally inactive and consequently stimulates HIF-1α-induced glucose flux and lactate production; (3) PHD2 is required for oncogenic H-ras^V12^-mediated metabolic reprogramming. Further characterization and understanding these distinctions would provide important evidence in the selective drug development targeting PHD2.

## Methods

### Reagents and antibodies

*N*-acetyl-l-cysteine (NAC), diamide, hydrogen peroxide (H_2_O_2_), MG132, PEG-catalase, *tert*-butyl hydroperoxide (T-hydro) and 2′–7′–dichrolodihydrofluorescein diacetate (DCF-DA) were purchased from Sigma Aldrich and Invitrogen. Antibodies against PHD2 (#4835), Proline 564-hydroxylated-HIF-1α (#3434), HIF-1α (#3716), phospho-ERK1/2 (#4376), and ERK1/2 (#4695) were purchased from Cell Signaling Technology. Anti-HA, Flag, PHD1, PHD3, FIH1 and β-tubulin were purchased from Sigma Aldrich and Santa Cruz Biotechnology.

### Cell culture, plasmids, and transfection

U2OS cells were cultured in Dulbecco’s modified Eagle’s medium (DMEM) containing 10% FBS and 25 mM glucose. Mammalian expressing pcDNA3-H-ras^V12^ and pcDNA3-H-ras^wt^ were gifted from Julian Downward (Addgene plasmid #39504 and #39503)^42^. Flag-tagged PHD1 (Addgene plasmid #36950), PHD2 (Addgene plasmid #36949) and PHD3 (Addgene plasmid #36951) lentiviral vectors were gifted from William Kaelin. HA-tagged PHD2 was provided from Jong-Wan Park (Seoul National University). Flag-tagged catalytic domain of PHD2 (181–426) was generated using a PCR-based mutagenesis with BamHI and NotI enzyme site contained specific oligonucleotides (Sense, 5′-AACGGGCAGACGAAGCCCCTG-3′; antisense, 5′-CTAGAAGACGTCTTTACCGACCGA-3′). Site-specific mutations of PHD2 (C283S, C302S, C323S, and C326S) were generated by PCR-based point mutagenesis (Stratagene). Mammalian expressing plasmids were transfected into U2OS cells using PolyFect (Qiagen) according to the manufacturer’s instructions. To generate stable cell lines, pcDNA3-H-ras^V12^- and pcDNA3-H-ras^wt^-transfected U2OS cells were incubated for 48 h, and were then cultured for 2 weeks with 700 μg/mL of G418.

### Western blotting and Co-immunoprecipitation

Total protein was prepared using a cell lysis buffer containing 1% IGEPAL, 150 mM NaCl, 50 mM Tris-HCl (pH 7.9), 10 mM NaF, 0.1 mM EDTA, and protease inhibitor cocktail. Protein samples were transferred into fresh tubes and then mixed with 2XSDS sample buffers containing β-ME (reducing) or without β-ME (non-reducing). For co-immunoprecipitation, HEK293T cells were transiently transfected with HA-tagged PHD2 and Flag-tagged PHD2, and then cells were further incubated for 24 h. Transfected cells were then incubated for 1 h in the absence or presence of hydrogen peroxide. Cell lysates were incubated with anti-Flag-conjugated beads for 1 h, and then complexes were washed with washing buffer (200 mM NaCl). Eluted proteins were subjected into SDS-PAGE, and then separated proteins were transferred onto a PVDF membrane (Millipore). Membranes were incubated with primary antibodies (1:1,000) in 5% bovine serum albumin containing 0.05% Tween-20 overnight at 4 °C, and HRP-conjugated secondary antibodies (1: 10,000) were incubated for 1 h at room temperature. Proteins levels were visualized using an ECL Prime kit (GE healthcare).

### Measurement of intracellular ROS levels

Intracellular hydrogen peroxide (H_2_O_2_) levels were determined using a DCF-DA and ROS-Glo^TM^ H_2_O_2_ assay kit purchased from Sigma Aldrich and Promega. Cells were incubated with Hank’s balanced slat solution containing 30 μmol/L of DCF-DA for 30 min. After incubation with DCF-DA, cells were detached in a trypsin-ehylenediaminetetraacetic acid (EDTA) solution and resuspended in phosphate-buffered saline (PBS) for fluorescence activated cell sorting (FACS) analysis. In addition, cultured cells were incubated with ROS-Glo^TM^ detection solution for 30 min, and then luminescence was measured using luminometer.

### Quantitative real-time PCR

Total RNA was isolated with TRIzol (Invitrogen), and 2 μg of total RNA was used for cDNA synthesis using a high capacity cDNA reverse transcription kit (Applied Biosystems). Quantitative PCR was performed using SYBR Green PCR Master Mix (Applied Biosystems). Experimental Ct values were normalized to 36B4, and relative mRNA expression was calculated versus 36B4 expression. The sequences of PCR primers (5′-3′) are CTTGGGACAGCAGCCTTAAT and CAAGCTGGACGTTAAAGGGA for PGK1; GGCATTGATGACTCCAGTGTT and ATGGAGCCCAGCAGCAA for GLUT1; ATGATGTCATTCCCACAATGGCCC and TGAACATTCTGGCTGGTGACAGGA for PDK1; ACCCAGTTTCCACCATGATT and CCCAAAATGCAAGGAACACT for LDHA; AGCTGCGCTGATAGACATCC and CTACCTCCACCATGCCAAGT for VEGF; GCGACGCAGCCTTTGAAT and CCACTCCAGCAGGGAAGGA for CA-IX; GGGATTGTCAACGTGCCTTA and CTGGGCAGCTATGTCATCAA for PHD1; GTTCCATTGCCCGGATAAC and CGACCTGATACGCCACTGTA for PHD2; CTGTTCCATTTCCCGGATAG and TCGACAGGCTGGTCCTCTAC for PHD3; GTGAAGGGTACAAGGGGGTG and ACATCTCAGCAACCCACACA for TBP.

### Luciferase assay

Hypoxia response element (HRE), VEGF promoter-luciferase, or β-gal plasmids were transiently transfected using a PolyFect (Qiagen) into pcDNA3-H-ras^wt^ or pcDNA3-H-ras^V12^ stably expressing U2OS cells. The cells were lysed and assayed for luciferase activity, and β-gal assays were performed for normalization of transfection efficiency.

### Glucose consumption and lactate production assay

Culture medium containing lactate and glucose levels were determined using assay kits (BioVision Research Products) following the manufacturer’s instructions. Briefly, cells were cultured in phenol-red free DMEM containing 10 mM glucose for 24 h, and then cultured medium was mixed with the reaction solution. After reaction, lactate and glucose concentrations were analysed at 450 and 570 nm as described previously^43^. All values were normalized to cellular protein concentration.

### *In vitro* hydroxylation assay

GST-tagged HIF-1α ODDD (oxygen-dependent degradation domain) and Flag-tagged PHD2 proteins were expressed in *Escherichia coli* BL21 and HEK293T cells, and then proteins were purified using GSH-affinity or Flag-affinity chromatography. For the *in vitro* HIF-1α hydroxylation assay, GST-tagged ODDD (50 ng) was incubated with purified Flag-tagged PHD2 at 30 °C for 30 min in a hydroxylation assay buffer^43^. The hydroxylation of HIF-1α was analysed by immunoblotting using an anti-Pro564(OH)-HIF-1α antibody.

### Statistical analysis

All data were analysed using the unpaired Student’s *t*-test for two experimental comparisons and one-way ANOVA with Tukey posttest for multiple comparisons. Data are represented as means ± standard deviations. *p* < 0.05 was considered statistically significant.

## Additional Information

**How to cite this article**: Lee, G. *et al.* Oxidative Dimerization of PHD2 is Responsible for its Inactivation and Contributes to Metabolic Reprogramming via HIF-1a Activation. *Sci. Rep.* **6**, 18928; doi: 10.1038/srep18928 (2016).

## Supplementary Material

Supplementary Figure
