# Supplementary material for: Oxidative Dimerization of PHD2 is Responsible for its Inactivation and Contributes to Metabolic Reprogramming via HIF-1α Activation
Source: Sci Rep. 2016 Jan 7;6:18928. doi: 10.1038/srep18928 (PMC4703963; doi:10.1038/srep18928)

Oxidative Dimerization of PHD2 is Responsible for its Inactivation and Contributes to Metabolic Reprogramming via HIF-1α Activation

Gibok Lee[1#], Hyung-Sik Won[2#], Yoon-Mi Lee[3#], Jae-Wan Choi[2], Taek-In Oh[1], Jeong-Hwa Jang[2], Dong-Kug Choi[2], Beong-Ou Lim[1], Young Jun Kim[1], Jong-Wan Park[4], Pere Puigserver[5], and Ji-Hong Lim[1*]

[1]Department of Biomedical Chemistry, College of Biomedical & Health Science, Konkuk University, Chungju 380-701, Chungbuk, Republic of Korea
[2]Department of Biotechnology, College of Biomedical & Health Science, Konkuk University, Chungju 380-701, Chungbuk, Republic of Korea
[3]Department of Food Bioscience, College of Biomedical & Health Science, Konkuk University, Chungju 380-701, Chungbuk, Republic of Korea
[4]Department of Pharmacology, Seoul National University, College of Medicine, Seoul 110-799, Republic of Korea
[5]Department of Cancer Biology, Dana-Farber Cancer Institute; Department of Cell Biology, Harvard Medical School, Boston, Massachusetts, USA

*Corresponding author:

Ji-Hong Lim, Ph.D.
E-mail: jhlim@kku.ac.kr

Supplementary Information

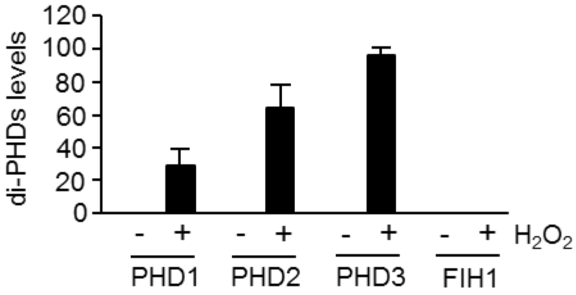

Supplementary Figure 1: Representative endogenous PHDs and FIH1 dimerization in U2OS cells

The dimeric form of PHDs and FIH1 levels were measured using immunoblotting and protein intensities were represented as bar graph using densitometry.

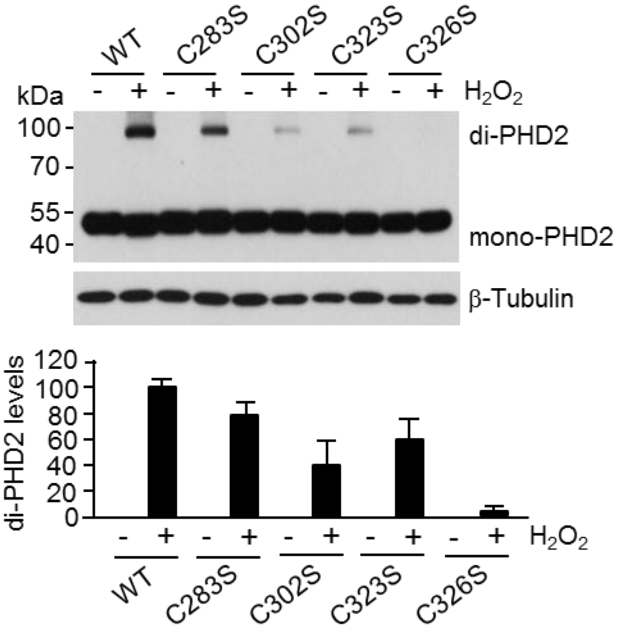

Supplementary Figure 2: Cys326 in the DSBH region is required for the oxidative dimerization of PHD2

Point mutated (C283S, C302S, C323S, and C326S) or wild-type HA-PHD2 were transiently transfected into U2OS cells, and then cells were incubated with 200 μM of H_2O_2 for 1 h. Total cell lysates were subjected into non-reducing SDS-PAGE, and then protein levels were measured by immunoblotting using anti-HA (*n*=3).

Supplementary Figure 3: Original images of western blots in Fig. 1A, Fig. 1C, Fig. 2B, Fig. 2C, Fig. 3A, Fig. 6B, Fig. 6C, Fig. 6D, Fig. 6E, Fig. 6G, and Supplementary Fig. 2.

Figure 1A (Non-reducing gel electrophoresis, IB: α-PHD2)

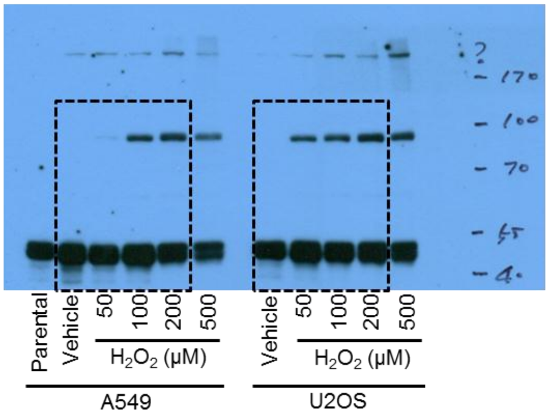

Figure 1A (Non-reducing gel electrophoresis, IB: α-PHD2)

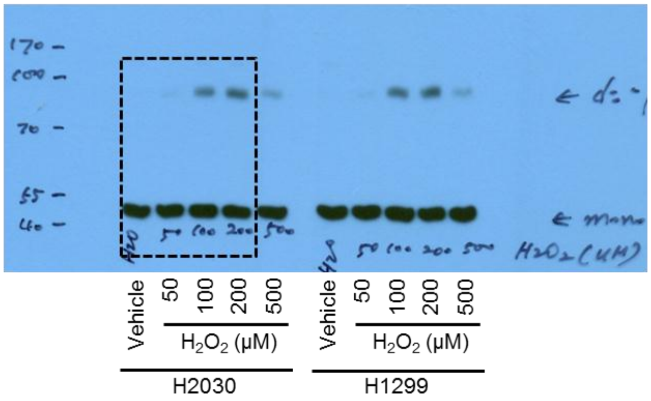

Figure 1C (Non-reducing or reducing gel electrophoresis, IB: α-PHD2)

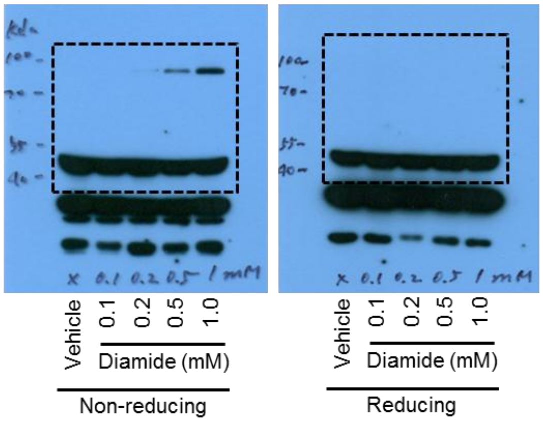

Figure 2B (Non-reducing gel electrophoresis, IB: α-PHD2)

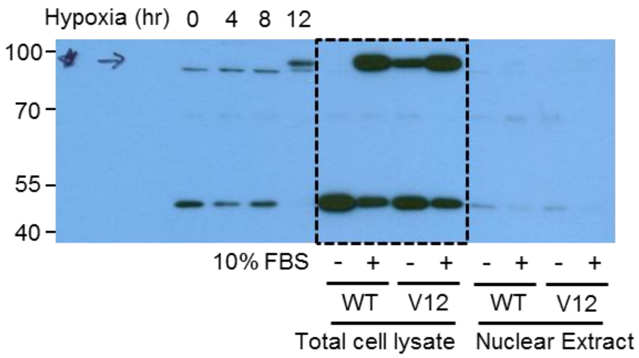

Figure 2C (Non-reducing gel electrophoresis, IB: α-PHD2)

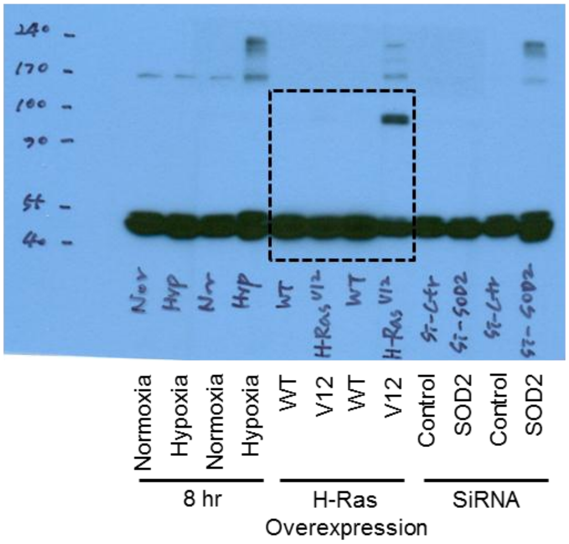

Figure 3A (Reducing or non-reducing gel electrophoresis after in vitro hydroxylation assay)

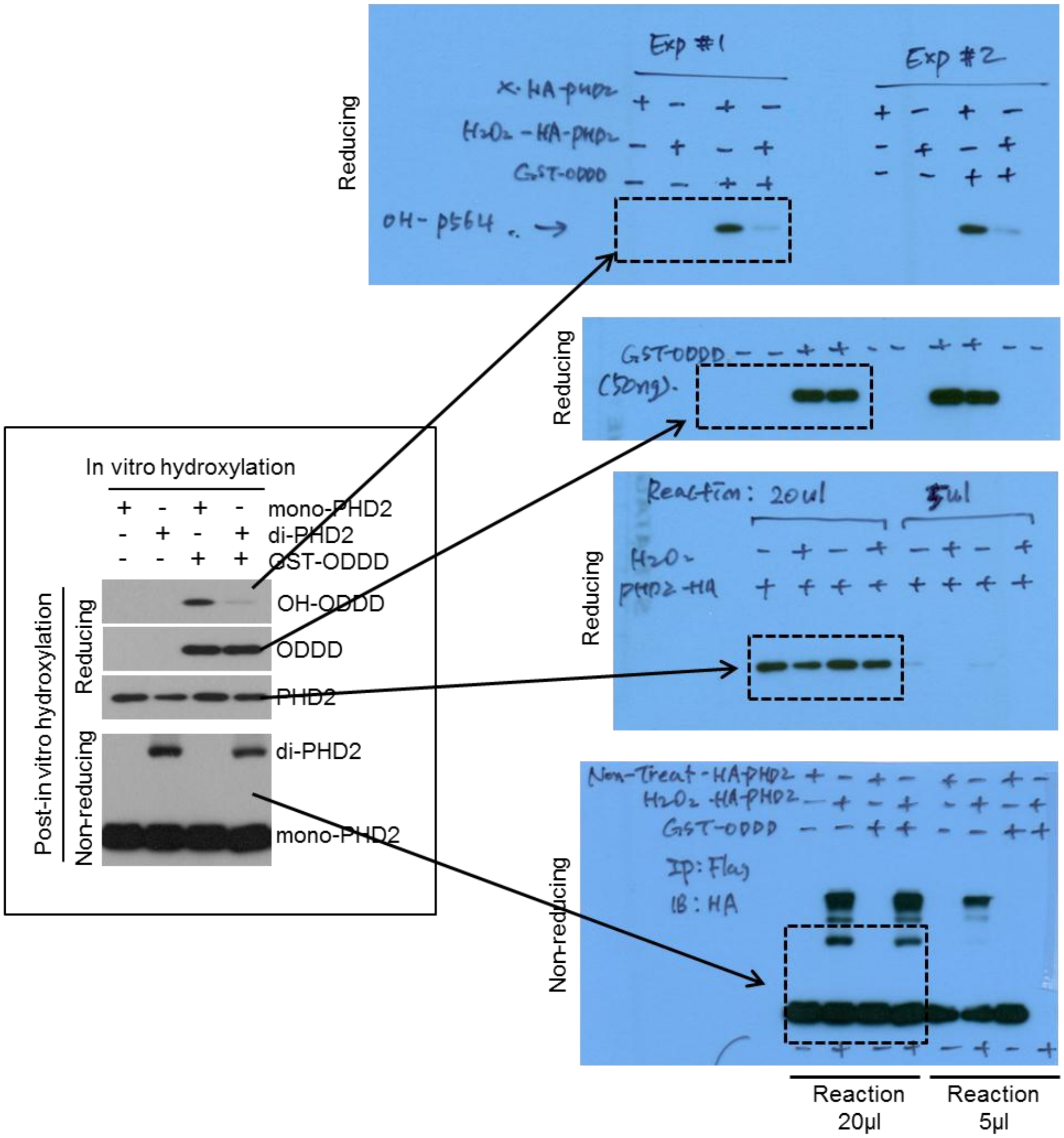

Figure 6B
(Gel electrophoresis with or without DTT, IB: α-Flag)

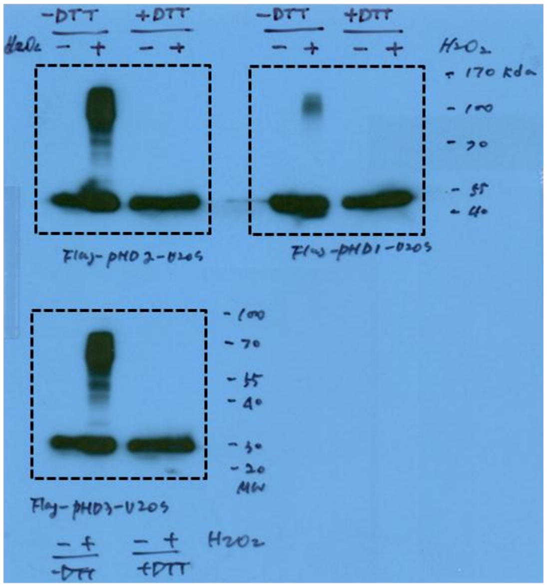

Figure 6C (Non-reducing gel)

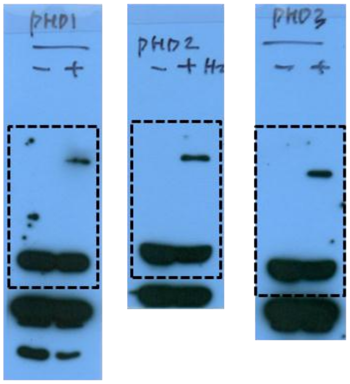

Figure 6D

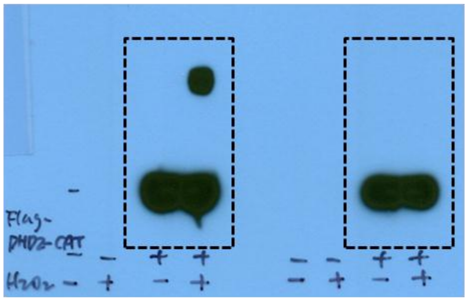

Figure 6E (Non-reducing)

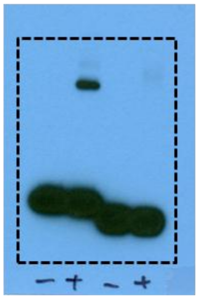

Figure 6G

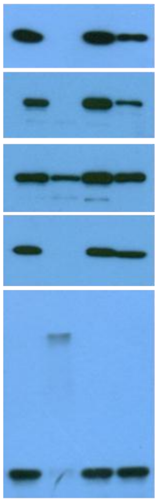

Supplementary Figure 2 (Non-reducing)

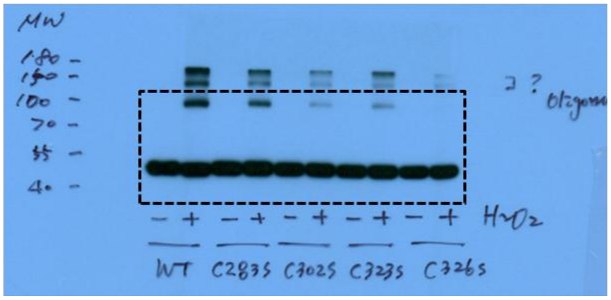

Supplement: Supplementary Figure [file srep18928-s1.pdf]
